# The live pig supply chain as reported by farmers in selected districts affected by African swine fever in Uganda, June and July 2022

**DOI:** 10.3389/fvets.2023.1234228

**Published:** 2023-07-31

**Authors:** Margaret Nawatti, John E. Ekakoro, David Singler, Krista Ochoa, Robinah Kizza, Dickson Ndoboli, Deo B. Ndumu, Eddie M. Wampande, Karyn A. Havas

**Affiliations:** ^1^Department of Political Science and Public Administration, College of Humanities and Social Sciences, Makerere University, Kampala, Uganda; ^2^Department of Public and Ecosystem Health, College of Veterinary Medicine, Cornell University, Ithaca, NY, United States; ^3^Central Diagnostic Laboratory, College of Veterinary Medicine, Animal Resources and Biosecurity, Makerere University, Kampala, Uganda; ^4^Department of Animal Health, Ministry of Agriculture, Animal Industry, and Fisheries, Entebbe, Uganda

**Keywords:** Uganda, supply chain, African swine fever, swine, pig, biosecurity

## Abstract

**Introduction:**

Uganda is a pork-producing country in East Africa. The African swine fever virus (ASFV) has had a devastating impact on the country’s pig industry. The movements of pigs and pork are a major pathway of spreading ASFV. This study was done to describe the live pig supply chain within and through districts that are impacted by African swine fever (ASF) in Uganda.

**Methods:**

A pig farmer survey in districts known to have ASFV was done using a semi-structured questionnaire available in English and two local languages. In total, 99 farmers were interviewed across five districts. Farmers were conveniently and purposively selected by local government veterinary officials. An online key informant survey was also used to validate farmer responses.

**Results:**

Most farmers interviewed in all districts reported to source and sell most of their pigs from within their district the farm was in, although there was variation by district and pig type. In relation to pig type, 89.7% of farmers sourced sows, 80.0% sourced boars, and 96.4% sourced weaned pigs from the district where the farm was located. As for sales, 91.3% of farmers sold sows, 92.7% sold boars, 91.9% sold weaned pigs, and 92.2% sold market pigs in the district where the farm was located. There was also variation to whom pigs were sold and sourced by pig type.

**Conclusion:**

This information is useful when planning the scale and focus of disease control programs based on animal movement. This study revealed that pig disease control programs can be targeted to smaller regions. Furthermore, there is a need for farmers and pig traders to be educated on and adhere to veterinary regulations of animal movement and good biosecurity practices to reduce disease spread when purchasing and selling pigs from known ASFV infected areas.

## 1. Introduction

African swine fever virus (ASFV) infection in pigs leads to a hemorrhagic, highly fatal, and contagious disease; exacerbating poverty, malnourishment, and food insecurity, and ultimately limiting the development of the swine industry (1–3). The ASFV is a large DNA arbovirus that is the lone member of the *Asfarviridae* family (4, 5) and has 24 recognized genotypes (6). ASFV is currently affecting pig industries throughout Africa, Europe, Asia, and Hispaniola (7), with genotype II being the primary viral variant found outside of the African continent (8). Historically, a variety of ASFV genotypes have impacted sub-Saharan Africa, Brazil, and other parts of the Caribbean (9).

Movement of infected pigs and pig products in one area to susceptible pigs in other areas is associated with ASFV spread in various locations (10–12). Animal movement has led to the spread of ASFV in many areas as it contributes to pig-to-pig spread that occurs reliably across many strains of differing virulence (13–17). For example, the legal live pig trade in China has been identified as a high-risk transmission pathway that moves ASFV from areas with disease to areas without (18). In Nigeria, it was shown that live animal markets where pig trading occurs are passageways for the spread of ASFV to new areas (12) as well. Various practices exist among Ugandan farmers that pose a threat of transmission, including obtaining of new live pigs without a health screening, and sharing of breeding boars among farms (19). It is likely that animal movement along the pig value chain in Uganda also can spread viruses such as ASFV.

There have been numerous studies that have established the presence of ASFV in all regions of Uganda (19–24). In addition, there is already some evidence that ASFV is spread through the supply chain in Uganda. In western Kenya and eastern Uganda, farmers sold pigs infected with ASFV to buyers who are not from the same area as the farmer (11), and this likely caused spread of disease. Indirect transmission is also a threat. The individuals involved in the pig supply chains contribute to the mechanical transmission of ASFV when they move from one farm to another to acquire pigs (11). Also, contaminated equipment and vehicles play a role in ASF transmission (10, 25). In Uganda, trucks are often rented (26, 27), and are not cleaned and disinfected (25) between uses, which can potentially spread disease to multiple groups of pigs in multiple locations.

Nonetheless, live pig movement in Uganda from areas known to have ASFV has not been extensively studied. Thus, understanding movement of pigs in a supply chain in these areas can elucidate how and where disease may be spread. The objective of this study was to understand the live pig supply chain across and through selected districts of Uganda that are impacted by African swine fever.

## 2. Materials and methods

### 2.1. Selection of study area and participants

**Figure 1:**
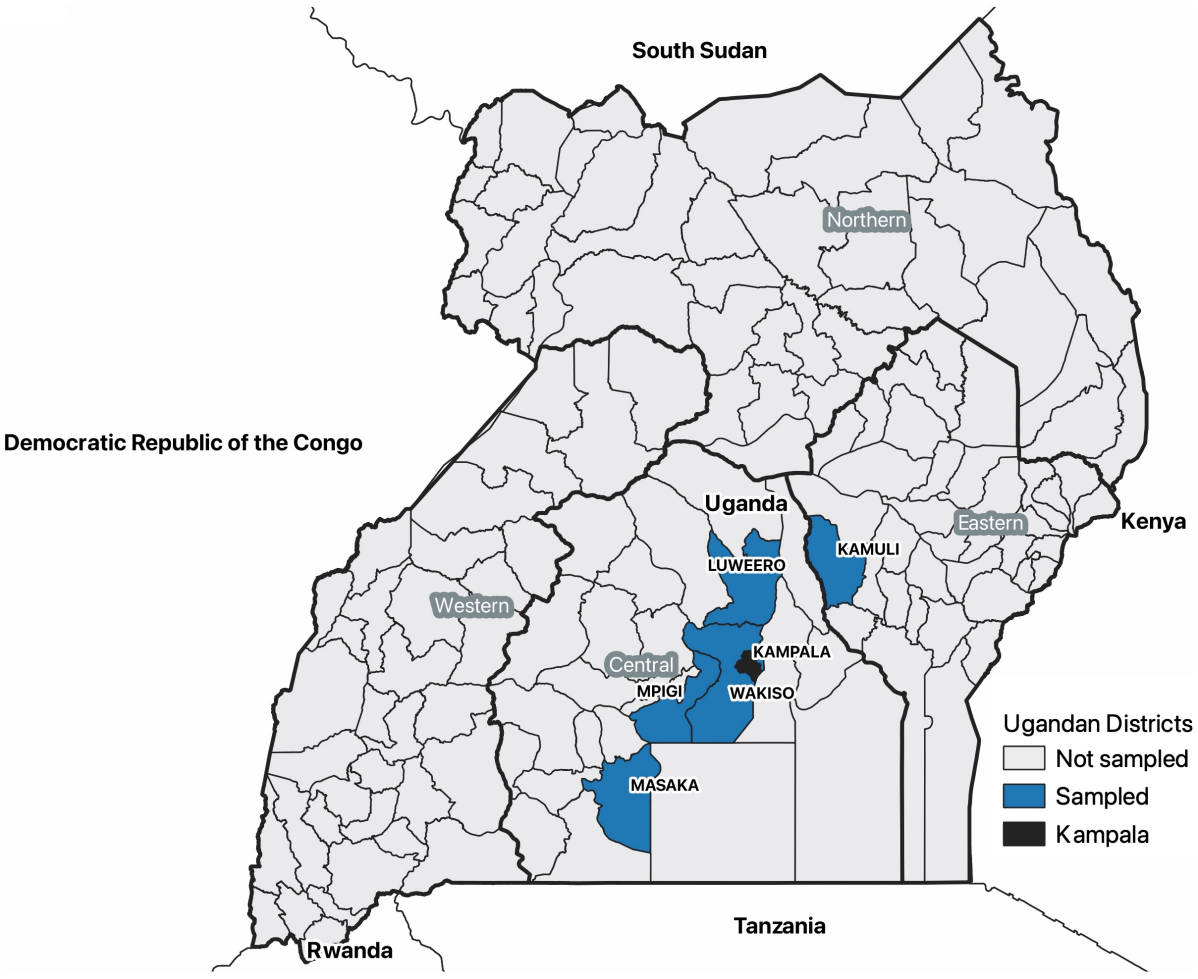
A map of Uganda showing the five selected districts most affected by African swine fever based on clinical and pathologic data collected from pigs slaughtered in the Kampala metropolitan area between May 2021 and June 2022. Farmers in these districts were included in a survey that summarized the live pig supply chain in June and July 2022. This map also indicates the location of the Kampala metropolitan area, administrative regions, and the countries surrounding Uganda.

The districts, which are local administrative units in Uganda, with the highest occurrence of clinical and pathologic signs for ASF in pigs assessed at Kampala metropolitan area slaughterhouses from May 2021 through June 2022 were enrolled in the study. To assess pigs, six abattoirs in the Kampala metropolitan area were purposively selected based on their annual slaughter rates. Sample sizes for each slaughterhouse were determined based on the abattoirs annual slaughter rates, and enough pigs were assessed to detect 200 ASFV pigs given a previous estimate of 11% prevalence (21). Pigs were then systematically sampled on randomly selected days of the month and each abattoir using a weighted sampling plan based on the annual slaughter rates. Clinical and pathologic signs of ASFV infection were gathered as well as data on pig type (local pigs, European pigs, cross-bred pigs), sex, and district of origin. The abattoir data collection tool was reviewed by African swine fever subject matter expert veterinarians, including veterinary pathologists in Uganda and the United States. Using this information, the districts that had the most pigs showing clinical signs and pathologic lesions commensurate for African swine fever were Wakiso, Masaka, Mpigi, Luweero, and Kamuli districts (Figure 1). This was confirmed by government veterinary officials as well.

A survey of farmers was completed to better understand pig farm biosecurity practices and supply chains in likely ASFV hotspots in June and July 2022. District-level government workers, particularly district animal health workers facilitated the farmer interviews. Participants were selected by local government veterinary officials to ensure a variety of farm sizes, locations, management systems, and farmer genders were captured. Sampling until saturation (28) of purposively selected farmer participants was done and data further evaluated using a key informant survey (19, 20). The key informant survey queried local experts on the topics farmers were asked about to confirm the responses. In total, 19 to 20 farmer participants were selected from each district totaling ninety-nine interviewed farmers. Farmers that were invited to participate had the option not to participate even after being selected by local officials. In addition, key informants were surveyed using an on-line questionnaire and they included district veterinary officers, para-veterinarians, animal husbandry officers, a veterinarian from a university, and animal scientists identified by the research team at Makerere University and by Dr. Ekakoro. Key informants were contacted via email invite them to take part in the survey and nineteen responded to our request.

### 2.2. Questionnaire development

The semi-structured questionnaire was developed by a team of Ugandan and American veterinarians and a public administration graduate student at Makerere University who is also a social worker. The questionnaire was pre-trialed among seven farmers in Kampala and Buikwe districts; these locations were not part of the study. The questionnaires were adjusted for greater clarity as recommended by participants in the pre-trial. The questionnaires were translated into Ugandan local languages, namely Luganda and Lusoga, by an individual with the Makerere University Institute of Languages. It was back translated to English by another individual and evaluated to ensure the translations properly presented the intended question. A unique identification number was given to each interview. The questionnaire recorded the date, district, gender of the farmer, and then asked questions regarding supply chain (source of pigs by type, where pigs were sold and to whom by type, source of boars used for breeding, and transportation) as well as questions about biosecurity. This manuscript focuses on the supply chain response. The key informant questionnaire was created by the same team, trialed among veterinarians at Makerere University, and was made available in English. Both questionnaires are available upon request.

### 2.3. Data collection

Farmer interviews were conducted at the farm by a female interviewer who spoke English, Lusoga, and Luganda. The interviewers were trained on the questionnaire prior to interviewing actual respondents and were accompanied by the local veterinary officials and students to make a mixed gendered team. The interviews were conducted in the preferred language of the farmer. Open ended responses were recorded in English. Interviews ranged from 30 min to 1 h on average. Farmers were reimbursed 20,000 Ugandan shillings (approximately USD 6) for their time. One individual declined the reimbursement. The questionnaire was loaded into Kobo Toolbox online and the Kobo Toolbox software (Kobo Organization, Cambridge, Massachusetts, United States) was downloaded onto a tablet computer into the Kobo collect app where the questionnaire responses could be recorded on-or off-line. The key informant questionnaire was administered in Qualtrics (Qualtrics, Provo, Utah, United States).

### 2.4. Data analysis

Data from Kobo Toolbox and Qualtrics were downloaded into Excel version 16.71 (Microsoft, Redmond, Washington, United States). A tri-lingual member of the research team back translated the Luganda and Lusoga language responses in the farmer questionnaire into English for analysis. Data were grouped into responses by pig type (female breeding pigs known as a sow or gilt, weaned pig, full grown or market pig, and boar). For this analysis, answers were combined and condensed for each pig type, but it did consider production system to verify data entry and farmer understanding. Data entry was reviewed to ensure accuracy and clarity among responses. Where data entry errors were suspected, two additional responses had to agree with one another and contradict with the expected date entry error to allow for changes. For example, if a respondent said that weaned pigs were purchased, but there were two further comments indicating weaned pigs were only born on the farm, the source of weaned pigs was changed to being born on farm. Further, the respondents that had boars or used boars for breeding their female pigs occasionally responded to questions meant for those that sold boar breeding services. They filled out information on where they sourced their boars but did not fill out information on to whom they sold boar services. These responses related to boar services only were discarded. The data was then summarized into frequency and percentages using Stata 16.1 IC (Stata Corp, College Station, Texas, United States). Trends were then compared.

### 2.5. Mapping

Mapping of districts was done using QGIS Firenze version 3.28.1.^1^ Shapefiles for Ugandan districts were obtained from the United Nations Human CR Operational Data Portal (Accessed March 30, 2023),^2^ for regions from the Office for the Coordination of Humanitarian Affairs (Accessed June 29, 2023),^3^ and for surrounding countries from ArcGIS (Accessed on July 5, 2023).^4^

## 3. Results

### 3.1. Farmer demographics and farm characteristics

Table 1 summarizes the characteristics of the farmers and their farms. A total of ninety-nine (99) respondents were interviewed and of these 56.6% (56/99) were male and 43.4% (43/99) female. Farm sizes varied. Most farmers, 39.4% (39/99), reared between four and 11 pigs, while only 3.0% (3/99) of farmers raised 1to 3 pigs or 41 to 50 pigs. Four production systems were identified. Farrow-to-finish production was done by 10.1% (10/99) of farmers. This is when pigs were born or farrowed on the farm and were raised to market weight or butchering weight. Only 6.1% (6/99) of farmers practiced farrow-to-wean production. This is when pigs were born or farrowed on farm and were sold to others to raise after they were weaned from the sow or female pig that gave birth to them. There were 36.4% (36/99) of farmers that provided boar breeding services. These farmers sold breedings by their boar to farmers that owned sows. Finally, 25.3% of farmers (25/99) practiced wean-to-finish production. They bought young pigs that were recently weaned from the sow and grew them until they were sold to be butchered or where market weight. Most of the farmers (81/99, 81.8%) practiced a combination of farrow-to-finish and farrow-to-wean production systems on their farms.

**Table 1:** Summary of farmer demographics and farm characteristics of farmers surveyed about their live pig supply chain in five Ugandan districts affected by African swine fever, June to July 2022.

	Kamuli		Luweero		Masaka		Mpigi		Wakiso		Overall	
	#	%	#	%	#	%	#	%	#	%	#	%
Farmers interviewed	20	100	20	100	20	100	19	100	20	100	99	100
**Gender of respondent**												
Male	16	80.0	9	45.0	10	50.0	7	37.0	14	70.0	56	56.6
Female	4	20.0	11	55.0	10	50.0	12	63.0	6	30.0	43	43.4
**Farm size**												
1–3	0	0.0	1	5.0	0	0.0	0	0.0	2	10.0	3	3.0
4–11	9	45.0	12	60.0	4	20.0	8	42.0	6	30.0	39	39.4
12–20	4	20.0	2	10.0	3	15.0	6	32.0	4	20.0	19	19.2
21–30	1	5.0	3	15.0	5	25.0	3	16.0	4	20.0	16	16.2
31–40	0	0.0	1	5.0	3	15.0	0	0.0	0	0.0	4	4.0
41–50	2	10.0	0	0.0	0	0.0	0	0.0	1	5.0	3	3.0
>50	4	20.0	1	5.0	5	25.0	2	10.0	3	15.0	15	15.2
**Farm labor**												
Family	12	60.0	18	90.0	9	45.0	13	68.0	11	55.0	63	63.6
Family and externally sourced	5	25.0	1	5.0	5	25.0	4	21.0	6	30.0	21	21.2
Externally sourced	3	15.0	1	5.0	6	30.0	2	11.0	3	15.0	15	15.2
**Production system**												
Farrow-to-finish	2	10.0	2	10.0	1	5.0	1	5.3	4	20.0	10	10.1
Farrow-to-wean	2	10.0	1	5.0	0	0.0	1	5.3	2	10.0	6	6.1
Boar service	7	35.0	6	30.0	7	35.0	8	42.0	8	40.0	36	36.4
Wean-to-finish	5	25.0	5	25.0	6	30.0	2	11.0	7	35.0	25	25.3
Farrow-to-finish & farrow-to-wean	16	80.0	16	80.0	19	95.0	16	84.0	14	70.0	81	81.8

### 3.2. Overall sourcing of live pigs

Table 2 summarized where the farmers sourced pigs. General sources were categorized as pigs born on the farm and obtained through external sources, such as other farmers, livestock markets, or through projects and non-governmental organizations (NGOs). Most farmers replaced sows from both pigs born on the farm and through external sources (41/95; 43.2%), with the next most common source being solely from pigs born on the farm (37/95; 38.9%). Overall, these responses suggested that most sows were replaced by pigs born on a farm. Farmers that purchased replacement sows purchased them from other farmers (53/58: 91.4%) in the same district (52/58; 89.7%). Boars used to sell breeding services were primarily obtained from outside the farm (17/36; 47.2%) and predominantly from fellow farmers (22/25; 88.0%) in the same district (20/25, 80.0%). Most farmers that raised weaned pigs raised pigs that were born on their farm (68/96; 70.8%). All farmers that obtained weaned pigs from outside the farm obtained them from fellow farmers (27/28; 96.4%) and/or from projects or NGOs (1/28; 3.6%). The purchased weaned pigs were from within the same district (27/28; 96.4%) and adjoining districts (2/28; 7.1%). Market pigs were grown from weaned pigs and were not purchased by farmers but were sold.

**Table 2:** Overall sourcing and selling of sows, boars, weaned and full-grown pigs from June – July 2022 in Kamuli, Luweero, Masaka, Mpigi and Wakiso districts, Uganda.

	Sows		Boars		Weaned pigs		Market pigs	
# of farmers surveyed = 99	*#*	%	*#*	%	*#*	%	*#*	%
	**Sourcing of pigs for the farm**							
**Source of pigs, *n***	**95**		**36**		**96**		**N/A**	
Born on farm	37	38.9	11	30.6	68	70.8	N/A	N/A
Some born on farm/ some obtained externally	41	43.2	8	22.2	25	26.0	N/A	N/A
All obtained externally	17	17.9	17	47.2	3	3.1	N/A	N/A
**External sources, *n***	**58**		**25**		**28**		**N/A**	
Livestock Market	0	0.0	1	4.0	0	0.0	N/A	N/A
Purchase from another farmer	53	91.4	22	88.0	27	96.4	N/A	N/A
Gift from project/NGO	8	13.8	3	12.0	1	3.6	N/A	N/A
Other^**^	2	3.4	0	0.0	0	0.0	N/A	N/A
**Districts of pig sourcing, *n***	**58**		**25**		**28**		**N/A**	
Same district	52	89.7	20	80.0	27	96.4	N/A	N/A
Adjoining districts	11	19.0	2	8.0	2	7.1	N/A	N/A
District without common border	7	12.0	6	24.0	0	0.0	N/A	N/A
**Method of transport, *n***	**58**		**25**		**28**		**N/A**	
Vehicle	43	74.1	13	52.0	8	28.6	N/A	N/A
Motorcycle	16	27.6	10	40.0	16	57.1	N/A	N/A
Walking	7	12.0	3	12.0	13	46.4	N/A	N/A
Bicycle	2	3.4	2	8.0	2	7.1	N/A	N/A
Wheel barrow	1	1.7	0	0.0	0	0.0	N/A	N/A
	**Sale of pigs from the farm**							
**Type of buyers, *n***	**69**		**55**		**86**		**90**	
Traders	34	49.3	12	21.8	14	16.3	56	62.2
Butchers	24	34.8	33	60.0	3	3.5	73	81.1
Other farmers	41	59.4	20	36.4	81	94.2	9	10.0
Family	6	8.7	4	7.3	17	19.8	8	8.9
Market	1	1.45	1	1.8	0	0.0	1	1.1
Local slaughter slab	0	0.0	4	7.3	0	0.0	18	20.0
Slaughter at the farm	2	2.9	2	3.6	0	0.0	3	3.3
Other^**^	2	2.9	2	3.6	12	14.0	0	0.0
**Districts of pig sales, *n***	**69**		**55**		**86**		**90**	
Same district	63	91.3	51	92.7	79	91.9	83	92.2
Adjoining districts	20	29.0	11	20.0	30	34.9	12	13.3
Districts without common border	20	29.0	15	27.3	24	27.9	25	27.8
Did not know	3	4.3	0	0.0	1	1.2	3	3.3

** Other external sources for sow sourcing included agricultural exhibitions and the government. For sales, farmers sold sows to the National Agriculture Advisory Services (NAADS) and to general organizations and weaned pigs to NAADS, schools, community members, as well as various organizations and projects. The bold values mean that a *n* number of farmers replied to that question.

There were differences in external sources by pig type. One farmer reported that they sourced boars from a livestock market (1/25; 4.0%) which was not reported for any other pig type. Two farmers (2/58; 3.4%) also obtained sows from the government and agricultural exhibitions, but the same was not reported for the boars and weaned pigs. More sows (11/58; 19.0%) were sourced from adjoining districts than boars (2/25; 8.0%) or weaned pigs (2/28; 7.1%). Farmers sourced sows (7/58; 12.0%) and boars (6/25; 24.0%) from districts without a common border with the farm’s district but did not source weaned pigs from distant districts. Transport of externally obtained pigs to the farm differed by pig type as well. Farmers mostly used vehicles to transport sows (43/58; 74.1%) and boars (13/25; 52.0%), but most weaned pigs were transported by motorcycle (16/28; 57.1%) and by walking the pig to the farm (13/28; 46.4%).

### 3.3. Overall sale of pigs

Table 2 also summarized where farmers sold pigs. Overall, 59.4% of sows (41/69) and 94.2% of weaned pigs (81/86) were sold to other farmers, and 91.3% (63/69) and 91.9% (79/86) were sold within the same district, respectively. On the other hand, 60.0% of boars (33/55) and 81.1% of market pigs (73/90) were sold to butchers with 92.7% (51/55) and 92.2% (83/90) sold within the same districts as the farm, respectively. Boars (4/55; 7.3%) and market pigs (18/90; 20.0%) were the only pig types reported to be sold to local slaughter slabs as well.

Comparison among sales by pig type revealed differences too. Sale to traders by farmers was highest for market pigs (56/90; 62.2%), then sows (34/69; 49.3%), and followed by boars (12/55; 21.8%). Weaned pigs were the least likely to be sold to traders (14/86; 16.3%). Sale to butchers was most common for market pigs (73/90; 81.1%), then boars (33/50; 60.0%), and, finally, sows (24/69; 34.8%). Weaned pigs (3/86; 3.5%) were the least likely pig type to be sold to butchers. As for sales to other farmers, 94.2% (81/86) of farmers sold weaned pigs, 59.4% (41/69) sold sows, and 36.4% sold boars (20/55), while only 10% (9/90) of market pigs were sold to other farmers. More farmers sold weaned pigs (17/86; 19.8%) to family members than any other pig type as well. A few farmers slaughtered sows (2/69, 2.9%), boars (2/55, 3.6%), and/or market pigs (3/90; 3.3%) at their farm rather than selling them. The government through the National Agricultural Advisory Services, schools, churches, as well as various projects and organizations bought sows (2/69; 2.9%), boars (2/55; 3.6%), and weaned pigs (12/86; 14.0%) too. As for district of sale beyond the district where the farm was located, more weaned pigs were sold to adjoining districts (30/86; 34.9%) than any other pig. While sow sales to districts that were not adjoining were the highest of all pig types (20/69; 29.0%). In addition, a few farmers did not know where sows (3/69; 4.3%), weaned pigs (1/86; 1.2%), or market pigs (3/90; 3.3%) went once sold.

### 3.4. Comparison of pig movements in the different districts

#### 3.4.1. Comparison of sourcing of live pigs

Supplementary Figure and Supplementary Table S1 through 5 described the movement of live pigs to and within different districts. When sourcing pigs, most farmers in all districts replaced their sows and weaned pigs through births on the farm or through a combination of births on the farm or external sources. Yet, sourcing of boars used to sell breeding services was variable across districts; 66.7% (4/6) of farmers in Luweero and 71.4% (5/7) of Masaka obtained boars solely from outside the farms compared to 42.8% (3/7) of farmers in Kamuli, 25.0% (2/8) in Mpigi, and 37.5% (3/8) in Wakiso. For pigs that were externally sourced, most farmers in all districts purchased pigs from other farmers in their home district. Yet, there was some variation across districts by pig type. All farmers in Wakiso sourced their pigs from within their home district. In Kamuli, 90% (9/10) of farmers sourced their sows from within the home district, 40% (4/10) from adjoining districts, and 10% (1/10) from districts without a common border. As for boars sourced by farmers in Masaka, 14.3% (1/7) obtained boars from adjoining districts and 71.4% (5/7) from districts without a common border. Finally, for weaned pigs, farmers in Kamuli, Mpigi, and Wakiso all sourced pigs from within their home district. Masaka had 100% (6/6) of farmers that sourced them from within the home district, but 16.7% of farmers (1/6) sourced from adjoining districts as well. In Luweero, 80% of farmers (4/5) sourced weaned pigs from within their home district and 20% (1/5) from adjoining districts. No farmers in any district sourced weaned pigs from districts without a common border with their home district. When looking at the figures, it is apparent that most sourcing occurred in closer proximity to the district of interest than far away. Although, farmers in Masaka sourced pigs from a district that was not part of a cluster around that district.

#### 3.4.2. Sale of pigs

Supplementary Figures S1–S5 and Supplementary Tables S1–S5 described the movement of live pigs from and within different districts. Sale of pigs differed by type as well as between districts. Most sows in Kamuli (12/16, 75.0%) and Mpigi (8/12, 66.7%) were sold to other farmers; in Luweero (6/9; 66.7%) and Masaka (11/17; 64.7%) were sold to traders; and in Wakiso were sold mainly to butchers (7/15; 46.7%) and other farmers (7/15; 46.7%). Weaned pigs were primarily sold to other farmers in all districts, with responses ranging from 81.25% (13/16) in Wakiso to 100% in Masaka (19/19) and Mpigi (17/17). Weaned pigs were also sold to traders, family, or other groups in all districts, except Mpigi which did not have farmers that sold weaned pigs to other groups. Other groups included the National Agriculture Advisory Services (NAADS), various organizations, and the community. Most market pigs were sold to butchers and then traders, except in Kamuli where market pigs were sold mostly to traders (12/16; 75.0%) then butchers (10/16; 62.5%). In Wakiso, the largest difference in sales between butchers (16/18; 88.9%) and traders (7/18; 38.9%) was found, and Wakiso farmers also reported lower sales to traders than other districts. Greater than 50% of farmers in all other districts reported selling market pigs to traders. Farmers in all districts sold boars most commonly to butchers and then to traders, except for Kamuli where 41.7% (5/12) of farmers sold both to butchers and traders.

Most farmers sold all pig types in their home district, but there was a great deal of variation between pig type for those sold outside of the home district. Kamuli and Masaka farmers had the highest export rates to other districts, while Wakiso had the lowest. Among Kamuli farmers, 43.75% (7/16) exported sows to adjoining districts and 25.0% (4/16) to districts without a common border; for boars it was 41.7% (5/12) and 25.0% (3/12), respectively; for weaned pigs it was 61.1% (11/16) and 38.9% (7/18), respectively; and for market pigs farmers exported 25.0% (4/16) to adjoining districts and 31.25% (5/16) to districts without a common border. Among Masaka farmers, 58.8% (10/17) of farmers sold sows and 73.7% (14/19) sold weaned pigs to both adjoining districts and those without a common border. For boars, 50.0% (5/10) of Masaka farmers sold to buyers in adjoining districts and 80.0% (8/10) sold to buyers in districts without a common border. Masaka farmers who sold market pigs sold 10.0% (2/20) to buyers in an adjoining district and 60.0% (12/20) to buyers in districts without common border. Farmers in Mpigi and Wakiso reported that they did not always know where their pigs went once sold as well. When looking at the figures it is apparent that most sales occurred in closer proximity to the district of interest than far away. Although, farmers in Kamuli and Masaka sold pigs to a district that was not part of a cluster around that given district.

#### 3.4.3. Key informants

Table 3 summarizes information given by key informants (KI) on how farmers source, sale and transport their pigs. They reported that it was very common for farmers to obtain sows by keeping those born on the farm (9/19; 47.4%), and, if sourced off farm, they were very commonly obtained from other farmers (10/19; 52.6%). Regarding weaned pigs, 47.4% (9/19) of KIs stated farmers sourced them from their own farms and 42.1% (8/19) of KIs stated farmers sourced them from other farmers. This was consistent with the farmers’ responses indicated above. They also reported that it was common for farmers to receive sows (10/19; 52.6%) and weaned pigs (36.8%; 7/19) from NGOs or as gifts from a project (10/19; 52.6%). Farmers reported receiving sows and weaned pigs as gifts as well. As for breeding boars, key informants reported that farmers obtained them commonly from pigs born on the farm (8/19; 42.1%), but it was not common for them to be received from NGOs or as gifts (14/19; 73.7%). Farmers reported obtaining most boars from external sources, and they did report obtaining them as gifts. The KI questionnaire asked about limited external sources of boars overall though. It was not common for any pig type to be sourced from livestock market.

**Table 3:** Summary of key informant responses regarding farmers’ sources, sale and transport of pigs by pig type in Uganda.

*n* = 19	Do not know	Uncommon	Common	Very common
Source of sows	#	#	#	#
Born on farm	0	1	6	9
From another farmer	0	0	6	10
Market	0	15	0	1
Gift from project/NGO	0	3	10	3
**Source of weaned pigs**				
Born on farm	0	0	7	9
From another farmer	0	0	8	8
Market	0	13	3	0
Gift from project/NGO	0	4	7	5
**Source of breeding boars**				
Born on farm	0	0	8	7
Market	0	11	5	0
Gift from project/NGO	0	14	9	3
**Sale of sows**				
Traders	0	4	7	3
Butchers	0	2	9	4
Other farmers	1	5	6	1
Family	0	6	9	0
Market	2	7	6	0
**Sale of weaned pigs**				
Traders	0	8	6	2
Butchers	0	13	3	0
Other farmers	0	1	7	8
Family	0	3	8	5
Market	2	9	4	1
**Sale of breeding boars**				
Traders	0	4	9	3
Butchers	1	5	8	2
Other farmers	1	3	6	4
Family	2	4	6	2
Market	1	4	8	2
**Sale of market pigs**				
Traders	0	0	4	11
Butchers	0	0	6	9
Other farmers	1	9	1	4
Family	2	10	2	1
Market	1	3	4	7
**Method of transport**	**Sow**	**Boar**	**Weaned pig**	**Market pig**
	#	#	#	#
Vehicle	12	11	9	11
Motorcycle	3	3	7	8
Walking	3	3	2	1
Wheelbarrow	0	1	0	0
Bicycle	0	0	4	3

KIs also provided input on the sale of pigs by farmers. They stated that sows were commonly sold to family members (9/19; 47.4%), butchers (9/19; 47.4%), and traders (7/19; 36.8%), which was consistent with the farmers’ responses above. KIs reported that weaned pigs were very commonly sold to other farmers (42.1%; 8/19) and commonly sold to family (42.1%; 8/19). The farmer responses stated weaned pigs were sold to other farmers, then family, and then traders. The breeding boars were commonly sold to traders (47.4%; 9/19), butchers (42.1%; 8/19), and at livestock markets (42.1%; 8/19). Yet, farmers rarely reported selling at livestock markets. Market pigs very commonly were sold to traders (57.9%; 11/19) and butchers (47.4%; 9/19), which was consistent with the farmers’ responses above. KIs reported that all pig types were predominately transported using vehicles and the least used transport was a wheelbarrow which was reported to be used when moving the breeding boars. Farmers reported using vehicles and then motorcycles to move sows and boars and using motorcycles, then walking, and lastly vehicles to move weaned pigs.

## 4. Discussion

This study was completed to understand the pig supply chain of live pigs across and through selected districts of Uganda that are impacted by African swine fever. Other similar studies have shown that cross border pig trade, transportation of infected pigs, and the frequent movements of pig traders could transmit ASFV across a range of distances (11, 27).

Pig farmer practices related to animal movements are a likely source of ASFV transmission within and between districts. We found that most pigs were sourced and sold in same district, but some farmers in the districts studied also sourced and sold pigs to districts that were adjoining or to districts without a common border to the home district. This was particularly true of breeding animals: boars and sows. This resulted in the possibility of a wide distribution of live pigs throughout the region, but most sales were in or clustered around the home district of farmers. This suggests that farmers are likely to export disease to other districts and an outbreak in one district may indicate infection in that district cluster as well. Farmers in Kamuli and Masaka reported the widest range of sales between districts and may be more likely to transmit disease long distances, but all districts sourced and sold to a group of districts that created a distinct cluster around the home district (Supplementary Figures S1–S5). These findings are consistent with other studies that showed pig movement across regions in Nigeria (12) and across the Ugandan and Kenyan borders (11) that transmitted diseases. Nonetheless, the most common external source of pigs for farmers was from other farmers in the same district, and this implies that pig farmers in the same district may also contribute to the ASFV transmission cycle. In Wakiso, all pigs were sourced from the home district, so maintenance of infection in this hotspot is likely within the district itself and less likely to be associated with imports of live pigs to the farms. Whether pigs are sourced from other districts or from within the home district, if buyers do not know the health history of the farm they are purchasing from and if they do not quarantine new pigs before bringing them into contact with their other pigs or housing, they may introduce disease. Quarantine and pre-purchase exams of the pig and the farm itself could reduce disease introductions to new locations.

Other actors engaged in the pig trade could contribute to disease spread as well, especially the butchers and traders as they buy pigs from farmers and move them to other areas. There are clear disease control requirements for purchase and movement of pigs, and it is believed that they are often ignored by individuals acting in the pig supply chain. The Ugandan Ministry of Agriculture, Animal Industry and Fisheries only allows the inter-district and inter-sub-district movement of healthy pigs (29), although the efficiency of the enforcement of this policy was not examined in this study. The uncertainty of some farmers in Masaka, Mpigi and Wakiso as to where pigs were sold could be due to the involvement of pig traders or that the individual interviewed took care of the pigs but were not engaged in the selling of the pigs and, thus, did not know. Since the output of the supply chain for butchers and traders is pork and pigs to slaughter (26), and swill is a common food source for swine (30), it will be important to consider swill feeding management in any districts that butchers or traders that buy from farmers sell pork or pigs to slaughter, respectively.

Another aspect that could contribute to inter-district disease spread was the use of vehicles to transport pigs. It is common that vehicles are rented in Uganda (16, 17) and are often not cleaned and disinfected between uses (15). Dirty vehicles pose a risk for indirect transmission of ASFV to pigs being transported and thus propagation of disease between sites. Sows and boars were commonly transported using vehicles in this study, and the key informants stated that all pigs were commonly moved using vehicles as well. Sows often stay for multiple years at a farm to produce piglets and boars are often used for breeding sows at one or multiple sites. ASFV is known to have various virulent forms (31) and pigs are known to survive infection (32, 33). Both sows and boars can lead to exposure of many other pigs to ASFV if they are exposed during transport and survive infection. Some farmers would also share vehicles with others and such an activity could further increase the risk of spreading the ASFV especially if some pigs being transported were infected.

There are key targets for disease control identified through this work. Since a significant number of pigs were sourced by retaining those that were born on farm then kept, increased biosecurity on feed, visitors, and transport could reduce the risk to those farms. Further, since most sales and sources cluster around the home district, local level control programs that target activities at the district level or within a cluster of districts could assist in ASFV control. This would allow targeting of interventions based on the farming practices by district and through support of farmers in a cluster of districts. This could potentially reduce the disease burden in heavily infected districts even if a national program is not feasible. The challenge will be managing sales into the district of pork or pigs from districts outside of the control program and managing economic losses to reduce sell offs or slaughter when outbreaks do occur. Nonetheless, the more scalable and customizable a control program from the national to the local level, the more success may be had.

Finally, because movements and transportation of pigs present risks of ASFV transmission, farmers, pig traders and other actors involved in the sourcing and selling of live pigs must adhere to veterinary regulations governing animal movement to reduce the risk of spread (29, 34). These include inspection of pigs prior to movement to ensure they are healthy. They should also take precautions to ensure biosecurity of the transportation used through cleaning and disinfecting and by limiting sharing with pigs of known health statuses where possible. The uncertainty of some farmers as to where pigs were sold could be due to the involvement of pig traders. Thus, accurate record-keeping and tracking of veterinary movement certificates is needed by pig traders as they are the final source of knowledge on pig movements. Finally, testing of ASFV suspect animals prior to movement or for diagnosis and control within districts would be more reliable than a health inspection alone as well. It would be ideal to conduct ongoing surveillance in districts to truly understand the disease burden as well.

This study had limitations. First, purposive and convenience sampling were used to conduct sampling until saturation, and the data were able to present themes and trends but is not representative. This sampling approach allows one to identify trends in a system (28, 35). These trends provide guidance to the most common and least common behaviors and activities among these farmers and give insights to pig movement in the region. Statistical analysis was not conducted since the purpose was to evaluate these trends. Yet, these trends were very revealing as to the general nature of the sourcing and sale of pigs within and between districts and how they cluster. Such key findings can be used to develop locally targeted control programs among farmers in connected districts. Second, clinical signs and pathologic lesions were used to determine districts of greatest ASFV burden. Syndromic methods were used as all diagnostic testing was incomplete at the time when the field work had to be completed. Yet, between the endemic nature of ASFV in Uganda, the commensurate clinical signs and pathologic lesions, and confirmation from the government veterinary officials as to the burden in these districts, we were confident that these districts would be appropriate for assessing supply chains in ASFV impacted areas even if some pigs were misclassified based on this approach.

## 5. Conclusion

The sourcing and sale of pigs from districts affected by ASFV pose a risk of ASFV transmission within the same district, adjoining districts, or districts without common borders especially if the biosecurity measures are not put into practice. Pig movements generally occurred within a district or nearby districts and among fellow farmers or to traders and butchers. Control programs could be targeted to connected districts rather than developed for national implementation and should include biosecurity components that address record-keeping of pig movements, transportation, evaluation of pig health, quarantine, and avoidance of pork in pig feed at a minimum. Finally, the use of required health certifications will improve on restricting the movement of pigs infected with ASFV.

## Data availability statement

The raw data supporting the conclusions of this article will be made available by the authors, without undue reservation.

## Ethics statement

The studies involving human participants were reviewed and approved by Collection of pig biodata: College of Veterinary Medicine, Animal Resources, and Biosecurity, Makerere University Higher Degrees Research Committee (reference number: SBLS.EWM.2020) Uganda National Council for Science and Technology (registration number: NS266ES). Cornell University’s Institutional Review Board (protocol number: 2012010020) Human Research Protection Office of the US Army Medical Research and Development Command (log number: A-21116). The biosecurity and supply chain survey: College of Veterinary Medicine, Animal Resources, and Biosecurity, Makerere University Higher Degrees Research Committee (reference number: SBLS.EWM.2020) Uganda National Council for Science and Technology (registration number: NS266ES). Cornell University’s Institutional Review Board (protocol number: IRB0010828) Human Research Protection Office of the US Army Medical Research and Development Command (log number: E03438.21). Written informed consent for participation was not required for this study in accordance with the national legislation and the institutional requirements. The animal study was reviewed and approved by College of Veterinary Medicine, Animal Resources, and Biosecurity, Makerere University Higher Degrees Research Committee (reference number: SBLS.EWM.2020) Uganda National Council for Science and Technology (registration number: NS266ES). Cornell University’s Institutional Animal Care and Use Committee (Protocol number: 2019–0108). US Army Medical Research and Development Command’s Animal Care and Use Review Office (protocol number: CT-2020-38). Written informed consent for participation was not obtained from the owners because These were slaughterhouse animals. The slaughterhouses agreed to participate in the study over the course of a year. So consent was not requested for each animal.

## Author contributions

MN, JE, KH, EW, DiN, and DeN contributed to the conception and design of the study as well as to questionnaire development. JE, EW, RK, MN, DS, KO, and KH contributed to data collection. KO and MN developed and organized the database. MN and KH conducted the statistical summary. KH provided geographic summaries. KH, DiN, and EW provided administrative oversight. MN and KH wrote the first draft of the manuscript. All authors contributed to the article and approved the submitted version.

## Funding

This research is sponsored by the U.S. Department of the Defense, Defense Threat Reduction Agency, under grant number HDTRA1-20-1-0007. The content of the information does not necessarily reflect the position or the policy of the federal government, and no official endorsement should be inferred.

## Conflict of interest

The authors declare that the research was conducted in the absence of any commercial or financial relationships that could be construed as a potential conflict of interest.

## Publisher’s note

All claims expressed in this article are solely those of the authors and do not necessarily represent those of their affiliated organizations, or those of the publisher, the editors and the reviewers. Any product that may be evaluated in this article, or claim that may be made by its manufacturer, is not guaranteed or endorsed by the publisher.

## Footnotes

^1^ qgis.org

^2^ https://data.unhcr.org/en/documents/details/83043

^3^ https://data.humdata.org/dataset/cod-ab-uga

^4^ https://hub.arcgis.com/datasets/africa::africa-countries/explore?location=-3.755856%2C19.067600%2C3.55
