# Supplementary material for: The live pig supply chain as reported by farmers in selected districts affected by African swine fever in Uganda, June and July 2022
Source: Front Vet Sci. 2023 Jul 31;10:1234228. doi: 10.3389/fvets.2023.1234228 (PMC10424570; doi:10.3389/fvets.2023.1234228)
Supplement: Supplementary file 1 [file Data_Sheet_1.pdf]

Supplementary Material

The live pig supply chain as reported by farmers in selected districts affected by African swine fever in Uganda, June and July 2022

Margaret Nawatti[1], John E Ekakoro[2], David Singler[2], Krista Ochoa[2], Robinah Kizza[3], Dickson Ndoboli[3], Deo B Ndumu[4], Eddie M Wampande[3], Karyn A Havas[2]

[1] Department of Political Science and Public Administration, College of Humanities and Social Sciences, Makerere University, Kampala, Uganda

[2] Department of Public and Ecosystem Health, College of Veterinary Medicine, Cornell University, Ithaca, New York, USA

[3]Central Diagnostic Laboratory, College of Veterinary Medicine, Animal Resources and Biosecurity, Makerere University, Kampala, Uganda

[4]Department of Animal Health, Ministry of Agriculture, Animal Industry, and Fisheries, Entebbe, Uganda

*** Correspondence:**
Karyn A Havas
kah47@cornell.edu

1 Supplementary Figures and Tables

1.1 Supplementary Tables

Table 1: Farmer information about sourcing and selling of weaned pigs, boars, sows, and market pigs in the Kamuli district of Uganda, June and July 2022

	Sows		Boars		Weaned Pigs		Market pigs	
	#	%	#	%	#	%	#	%
	SOURCING OF PIGS FOR THE FARM							
**Source of pigs, n**	**18**		**7**		**19**		**N/A**	
Born on farm	8	44.4	3	42.8	13	68.4	N/A	N/A
Some born on farm/ some obtained externally	6	33.3	1	14.3	5	26.3	N/A	N/A
All obtained externally	4	22.2	3	42.8	1	5.3	N/A	N/A
**External sources, n**	**10**		**4***		**6**		**N/A**	
Purchase from another farmer	10	100.0	3	100.0	6	100	N/A	N/A
Agricultural exhibitions	1	10.0	0	0.0	0	0.0	N/A	N/A
**Districts of pig sourcing, n**	**10**		**4**		**6**			
Same district	9	90.0	4	100.0	6	100	N/A	N/A
Adjoining districts	4	40.0	1	25.0	0	0.0	N/A	N/A
District without common border	1	10.0	0	0.0	0	0.0	N/A	N/A
**Method of transport**	**10**		**4**		**6**			
Vehicle	8	80.0	1	25.0	3	50.0	N/A	N/A
Motorcycle	4	40.0	2	50.0	5	83.3	N/A	N/A
Walking	1	10.0	1	25.0	1	16.7	N/A	N/A
Bicycle	0	0.0	1	25.0	0	0.0	N/A	N/A
	SALE OF PIGS FROM FARM							
**Type of buyers, n**	**16**		**12**		**18**		**16**	
Traders	9	56.25	5	41.7	3	16.7	12	75.0
Butchers	3	18.75	5	41.7	1	5.6	10	62.5
Other farmers	12	75.0	7	58.3	17	94.4	2	12.5
Family	2	12.5	0	0.0	1	5.6	2	12.5
Market	0	0.0	0	0.0	0	0.0	0	0.0
Local slaughter slab	0	0.0	0	0.0	0	0.0	2	12.5
Slaughter on farm	2	12.5	0	0.0	0	0.0	1	6.25
Other**	0	0.0	0	0.0	5	27.8	0	0.0
**Districts of pig sale**	**16**		**12**		**18**		**16**	
Same district	15	93.75	12	100	16	88.9	13	81.25
Adjoining districts	7	43.75	5	41.7	11	61.1	4	25.0
District without common border	4	25.0	3	25.0	7	38.9	5	31.25

* One individual reported that they externally sourced weaned pigs but gave no further information about from where. The denominator used to calculate proportions was three and not four.

** Other groups for weaned pig sales include various organizations, church, community and the National Agriculture Advisory Services. Other for boar sales are community slaughter slabs.

Table 2: Farmer information about sourcing and selling of weaned pigs, boars, sows, and market pigs in the Luweero district of Uganda, June and July 2022

	Sows		Boars		Weaned Pigs		Market pigs	
	#	%	#	%	#	%	#	%
	SOURCING OF PIGS FOR THE FARM							
**Source of pigs, n**	**19**		**6**		**19**		**N/A**	
Born on farm	6	31.6	1	16.7	14	73.7	N/A	N/A
Some born on farm/ some obtained externally	9	47.4	1	16.7	4	21.1	N/A	N/A
All obtained externally	4	21.1	4	66.7	1	5.3	N/A	N/A
**External sources, n**	**13**		**5**		**5**		**N/A**	
Purchase from another farmer	11	84.6	5	100.0	5	100.0	N/A	N/A
Gift from project/NGO	3	23.1	1	20.0	0	0.0	N/A	N/A
**Districts of pig sourcing, n**	**13**		**5**		**5**		**N/A**	
Same district	11	84.6	4	80.0	4	80.0	N/A	N/A
Adjoining districts	2	15.4	0	0.0	1	20.0	N/A	N/A
District without common border	2	15.4	1	20.0	0	0.0	N/A	N/A
**Method of transport,**	**13**		**5**		**5**		**N/A**	
Vehicle	7	53.8	2	40.0	1	20.0	N/A	N/A
Motorcycle	4	30.8	2	40.0	2	40.0	N/A	N/A
Walking	2	15.4	1	20.0	4	80.0	N/A	N/A
Bicycle	1	7.7	1	20.0	0	0.0	N/A	N/A
	SALE OF PIGS FROM FARM							
**Type of buyers, n**	**9**		**12**		**16**		**18**	
Traders	6	66.7	2	16.7	4	25.0	14	77.8
Butchers	2	22.2	4	33.3	0	0.0	15	83.3
Other farmers	4	44.4	5	41.7	15	93.75	2	11.1
Family	0	0.0	2	16.7	4	25.0	3	16.7
Market	0	0.0	0	0.0	0	0.0	0	0.0
Local slaughter slab	0	0.0	1	8.3	0	0.0	6	33.3
Other**	0	0.0	1	8.3	1	6.25	0	0.0
**Districts of pig sale, n**	**9**		**12**		**16**		**18**	
Same district	8	88.9	9	75.0	16	100.0	18	100.0
Adjoining district	1	11.1	1	8.3	3	18.75	2	11.1
District without common border	1	11.1	1	8.3	1	6.25	4	22.2

** Other groups include schools as well as farms (for boars).

Table 3: Farmer information about sourcing and selling of weaned pigs, boars, sows, and market pigs in the Masaka district of Uganda, June and July 2022

	Sows		Boars		Weaned Pigs		Market pigs	
	#	%	#	%	#	%	#	%
	SOURCING PIGS FOR THE FARM							
**Source of pigs, n**	**20**		**7**		**20**		**N/A**	
Born on farm	5	25.0	0	0.0	14	70.0	N/A	N/A
Some born on farm/ some obtained externally	13	65.0	2	28.6	6	30.0	N/A	N/A
All obtained externally	2	10.0	5	71.4	0	0.0	N/A	N/A
**External sources, n**	**15**		**7**		**6**		**N/A**	
Purchase from another farmer	13	86.7	5	71.4	6	100	N/A	N/A
Gift from project/NGO	3	20.0	2	28.6	0	0.0	N/A	N/A
**Districts of pig sourcing, n**	**15**		**7**		**6**		**N/A**	
Same district	13	86.7	3	42.9	6	100	N/A	N/A
Adjoining districts	3	20.0	1	14.3	1	16.7	N/A	N/A
District without common border	3	20.0	5	71.4	0	0.0	N/A	N/A
**Method of transport, n**	**15**		**6**		**6**		**N/A**	
Vehicle	13	86.7	6	100.0	1	16.7	N/A	N/A
Motorcycle	3	20.0	1	16.7	4	66.7	N/A	N/A
Walking	1	6.7	0	0.0	2	33.3	N/A	N/A
	SALE OF PIGS FROM FARM							
**Type of buyers, n**	**17**		**10**		**19**		**20**	
Traders	11	64.7	1	10.0	3	15.8	13	65.0
Butchers	5	29.4	7	70.0	0	0.0	17	85.0
Other farmers	10	58.8	3	30.0	19	100.0	2	10.0
Family	2	11.8	1	10.0	4	21.05	1	5.0
Market	0	0.0	0	0.0	0	0.0	0	0.0
Local slaughter slab	0	0.0	0	0.0	0	0.0	2	10.0
Other**	1	5.9	1	10.0	4	21.05	0	0.0
**Districts of pig sales, n**	**17**		**10**		**19**		**20**	
Same district	16	94.1	9	90.0	17	89.5	20	100.0
Adjoining district	10	58.8	5	50.0	14	73.7	2	10.0
District without common border	10	58.8	8	80.0	14	73.7	12	60.0
Did not know	0	0.0	0	0.0	0	0.0	1	5.0

** Other groups for sow sales include the National Agriculture Advisory Services (NAADS), for boars they are projects and organizations, and for weaned pigs they include various projects and organizations as well as NAADS.

Table 4: Farmer information about sourcing and selling of weaned pigs, boars, sows, and market pigs in the Mpigi district of Uganda, June and July 2022

	Sows		Boars		Weaned Pigs		Market pigs	
	#	%	#	%	#	%	#	%
	SOURCING OF PIGS FOR THE FARM							
**Source of pigs, n**	**18**		**8**		**19**		**N/A**	
Born on farm	8	44.4	3	37.5	16	84.2	N/A	N/A
Some born on farm/ some obtained externally	6	33.3	3	37.5	2	10.5	N/A	N/A
All obtained externally	4	22.2	2	25.0	1	5.3	N/A	N/A
**External sources, n**	**10**		**5**		**3**		**N/A**	
Livestock Market	0	0.0	1	20.0	0	0.0	N/A	N/A
Purchase from another farmer	10	100.0	5	100.0	3	100.0	N/A	N/A
Gift from project/NGO	1	10.0	0	0.0	0	0.0	N/A	N/A
**Districts of pig sourcing, n**	**10**		**5**		**3**		**N/A**	
Same district	9	90.0	5	100.0	3	100.0	N/A	N/A
Adjoining districts	2	20.0	0	0.0	0	0.0	N/A	N/A
District without common border	1	10.0	0	0.0	0	0.0	N/A	N/A
**Method of transport, n**	**10**		**5**		**3**		**N/A**	
Vehicle	7	70.0	2	40.0	2	66.7	N/A	N/A
Motorcycle	3	30.0	3	60.0	2	66.7	N/A	N/A
Walking	2	20.0	1	20.0	2	66.7	N/A	N/A
Bicycle	1	10.0	0	0.0	2	66.7	N/A	N/A
	SALE OF PIGS FROM FARM							
**Type of buyers, n**	**12**		**9**		**17**		**18**	
Traders	1	8.3	2	22.2	3	17.65	10	55.6
Butchers	7	58.3	7	77.8	1	5.9	15	83.3
Other farmers	8	66.7	2	22.2	17	100.0	2	11.1
Family	0	0.0	1	11.1	4	23.5	1	5.6
Market	1	8.3	1	11.1	0	0.0	1	5.6
Local slaughter slab	0	0.0	1	11.1	0	0.0	7	38.9
Slaughter at the farm	0	0.0	1	11.1	0	0.0	0	0.0
**Districts of pig sales, n**	**12**		**9**		**17**		**18**	
Same district	10	83.3	9	100.0	15	88.2	16	88.9
Adjoining district	0	0.0	0	0.0	2	11.8	2	11.1
District without common border	4	33.3	3	33.3	2	11.8	7	38.9
Did not know	1	8.3	0	0.0	0	0.0	2	11.1

Table 5: Farmer information about sourcing and selling of weaned pigs, boars, sows, and market pigs in the Wakiso district of Uganda, June and July 2022

	Sows		Boars		Weaned Pigs		Market pigs	
	#	%	#	%	#	%	#	%
	SOURCING OF PIGS FOR THE FARM							
**Source of pigs, n**	**20**		**8**		**19**		**N/A**	
Born on farm	10	50.0	4	50.0	11	57.9	N/A	N/A
Some born on farm/ some obtained externally	7	35.0	1	12.5	8	42.1	N/A	N/A
All obtained externally	3	15.0	3	37.5	0	0.0	N/A	N/A
**External sources, n**	**10**		**4**		**8**		**N/A**	
Purchase from another farmer	9	90.0	4	100	7	87.5	N/A	N/A
Gift from project/NGO	1	10.0	0	0.0	1	12.5	N/A	N/A
From government	1	10.0	0	0.0	0	0.0	N/A	N/A
**Districts of pig sourcing, n**	**10**		**4**		**8**		**N/A**	
Same district	10	100	4	100	8	100.0	N/A	N/A
**Method of transport, n**	**10**		**4**		**8**		**N/A**	
Vehicle	8	80.0	2	50.0	1	12.5	N/A	N/A
Motorcycle	2	20.0	2	50.0	3	37.5	N/A	N/A
Walking	1	10.0	0	0.0	4	50.0	N/A	N/A
Wheel barrow	1	10.0	0	0.0	0	0.0	N/A	N/A
	SALE OF PIGS FROM FARM							
**Type of buyers, n**	**15**		**12**		**16**		**18**	
Traders	7	46.7	2	16.7	1	6.25	7	38.9
Butchers	7	46.7	10	83.3	1	6.25	16	88.9
Other farmers	7	46.7	3	25.0	13	81.25	1	5.6
Family	2	13.3	0	0.0	4	25.0	1	5.6
Market	0	0.0	0	0.0	0	0.0	0	0.0
Local slaughter slab	0	0.0	2	16.7	0	0.0	1	5.6
Slaughter at the farm	0	0.0	1	8.3	0	0.0	2	11.1
Other**	1	6.7	0	0.0	2	12.5	0	0.0
**Districts of pig sales, n**	**15**		**12**		**16**		**18**	
Same district	14	93.3	12	100.0	15	93.75	18	100.0
Adjoining district	2	13.3	0	0.0	0	0.0	1	5.6
District without common border	1	7.1	0	0.0	0	0.0	0	0.0
Did not know	2	13.3	0	0.0	1	6.25	0	0.0

** Other groups include general organizations for sale of sows and general organizations and the community for weaned pigs.

1.2 Supplementary Figures

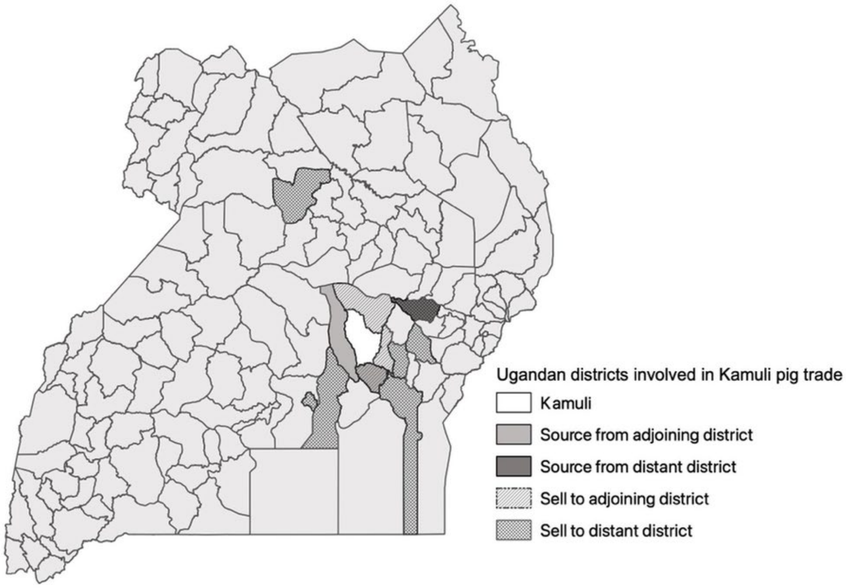

Figure 1: A map showing districts to and from which farmers in the Kamuli district of Uganda sell and source their live pigs based on a survey conducted between June and July 2022. If any farmer in Kamuli sourced or sold from a district it was indicated in the map. Colors were used to indicate adjoining and distant districts sourced from, and textures indicated adjoining and distant districts sold to; there was overlap between districts sourced from and sold to as well.

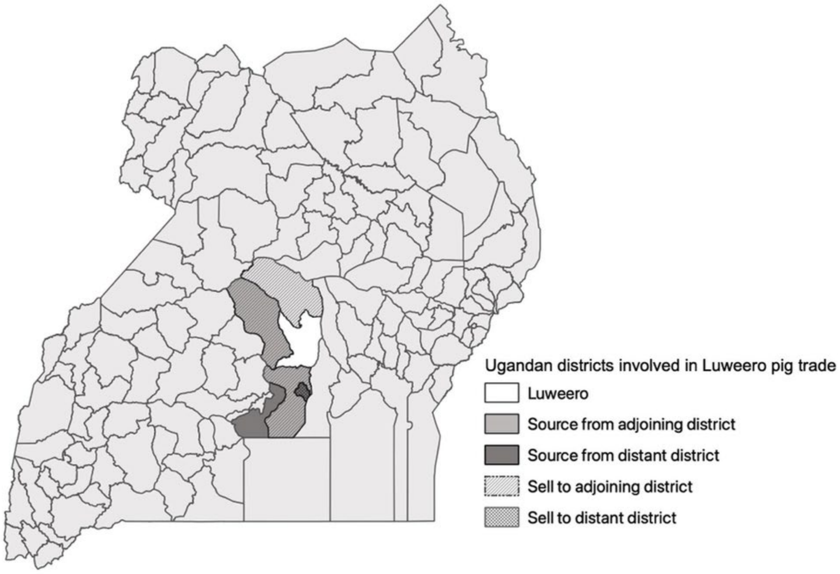

Figure 2: A map showing districts to and from which farmers in the Luweero district of Uganda sell and source their live pigs based on a survey conducted between June and July 2022. If any farmer in Luweero sourced or sold from a district it was indicated in the map. Colors were used to indicate adjoining and distant districts sourced from, and textures indicated adjoining and distant districts sold to; there was overlap between districts sourced from and sold to as well.

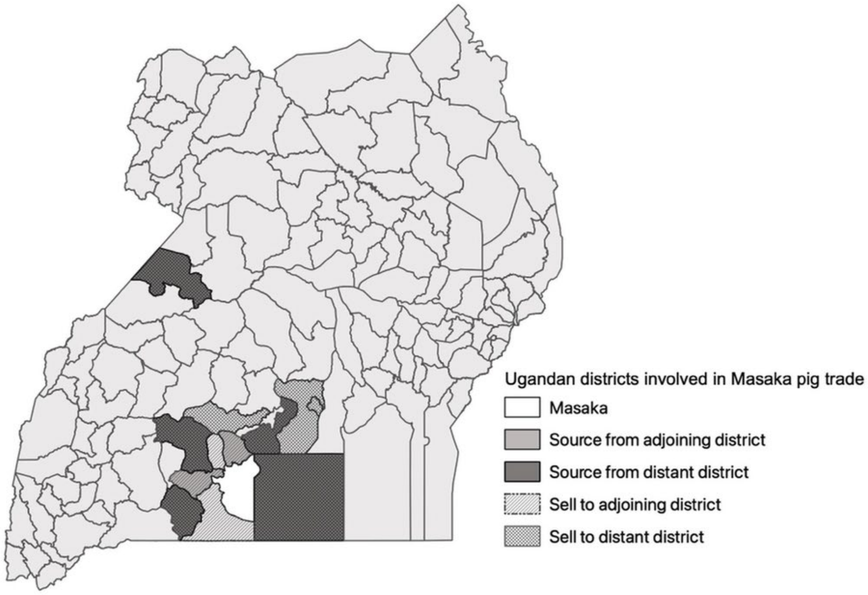

Figure 3: A map showing districts to and from which farmers in the Masaka district of Uganda sell and source their live pigs based on a survey conducted between June and July 2022. If any farmer in Masaka sourced or sold from a district it was indicated in the map. Colors were used to indicate adjoining and distant districts sourced from, and textures indicated adjoining and distant districts sold to; there was overlap between districts sourced from and sold to as well.

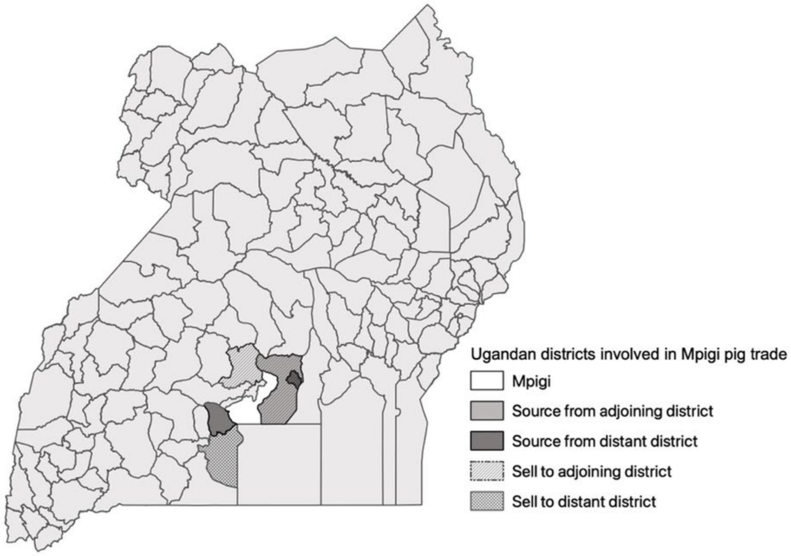

Figure 4: A map showing districts to and from which farmers in the Mpigi district of Uganda sell and source their live pigs based on a survey conducted between June and July 2022. If any farmer in Mpigi sourced or sold from a district it was indicated in the map. Colors were used to indicate adjoining and distant districts sourced from, and textures indicated adjoining and distant districts sold to; there was overlap between districts sourced from and sold to as well.

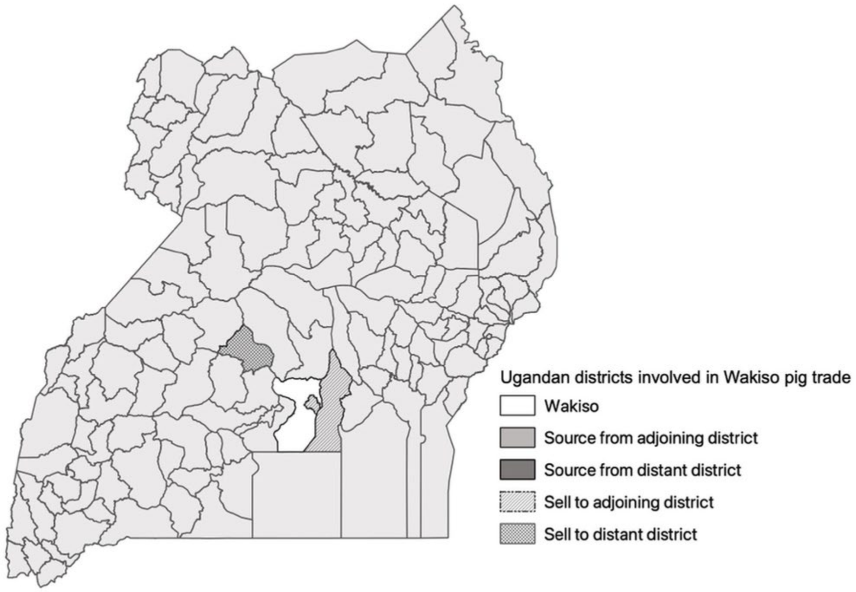

Figure 5: A map showing districts to and from which farmers in the Wakiso district of Uganda sell and source their live pigs based on a survey conducted between June and July 2022. If any farmer in Wakiso sourced or sold from a district it was indicated in the map. Colors were used to indicate adjoining and distant districts sourced from, and textures indicated adjoining and distant districts sold to.
